# Effleurage massage as a non-pharmacological intervention for labor pain management among primigravida mothers

**DOI:** 10.6026/973206300213550

**Published:** 2025-10-31

**Authors:** Soma Bepari, Siva Subramanian N, Mahalakshmi B

**Affiliations:** 1 Department of Obstetrics & Gynecological Nursing, Nootan College of Nursing, Sankalchand Patel University, Visnagar, Gujarat - 384315, India; 2 Department of Psychiatric Nursing, Nootan College of Nursing, Sankalchand Patel University, Visnagar, Gujarat - 384315, India; 3 Department of Paediatric Nursing, Nootan College of Nursing, Sankalchand Patel University, Visnagar ,Gujarat - 384315, India

**Keywords:** Effleurage massage, labor pain, primigravida, non-pharmacological intervention, pain management, quasi-experimental study, intrapartum care

## Abstract

Labor pain is one of the most intense physiological pains experienced by primigravida mothers. Therefore, it is of interest to assess the effectiveness of effleurage back massage in reducing labor pain among 180 primigravida women in selected hospitals of Bharuch, Gujarat. Hence, Participants were divided into experimental (n=90) and control (n=90) groups. The experimental group received effleurage massage during active labor, showing a significant reduction in pain scores compared to the control group (p < 0.001). Data shows that effleurage massage is a safe, cost-effective and evidence-based non-pharmacological intervention for labor pain management. Integration of effleurage massage into routine intrapartum care is recommended to improve maternal comfort.

## Background:

Labor pain in primigravida women, considered one of the most severe physiological discomforts due to uterine contractions, cervical changes and fetal descent, can lead to significant emotional distress, anxiety and increased requests for pharmacological interventions or cesarean delivery driven by fear of pain [1]. While techniques such as epidural anesthesia are widely used for relief, they bring about side effects including hypotension, prolonged labor and negative neonatal outcomes, making non-pharmacological methods attractive for many [2]. Growing evidence supports the value of non-pharmacological labor pain management strategies such as massage, hydrotherapy, breathing exercises and positioning [3]. Among these, effleurage massage-a gentle, rhythmic stroking applied typically to the lower back or abdomen-stands out as an accessible and easily administered intervention [4]. The theoretical basis is the gate control theory of pain, which posits that sensory input from touch can inhibit pain signal transmission in the central nervous system, thereby reducing pain perception and a recent systematic review confirmed that non-pharmacological techniques such as effleurage massage effectively mitigate pain and decrease the need for pharmacological interventions during labor [5]. Clinical studies have consistently demonstrated reductions in subjective pain scores (such as the Visual Analogue Scale and Numeric Pain Rating Scale) following effleurage massage compared to routine care or counter-pressure techniques [6]. For example, a 2023 quasi-experimental study revealed that women receiving effleurage massage experienced a significant decrease in pain intensity during labor, leading to higher maternal satisfaction and improved birthing experiences [7]. Meta-analyses have confirmed that massage therapy significantly reduces pain intensity across all stages of labor, with the greatest effect observed during the active and transitional phases, while techniques such as effleurage additionally promote maternal autonomy, comfort and emotional confidence-benefits that are particularly valuable for primigravida mothers with no prior delivery experience [8]. Integrating this cost-effective, evidence-based approach can substantially improve maternal comfort, satisfaction and potentially clinical outcomes during childbirth. Therefore, it is of interest to evaluate the effectiveness of effleurage massage in managing labor pain among primigravida mothers.

## Methodology:

## Study design and setting:

This quasi-experimental, non-randomized control group pre-test post-test design study was conducted in selected public and private hospitals in Bharuch, Gujarat, where institutional deliveries routinely occur. Labor rooms in these hospitals provided the setting for administering the intervention and collecting data.

## Participants and sampling:

The study targeted primigravida women aged 18 to 35 years in the active phase of labor (cervical dilation ≥4 cm) with singleton pregnancies and vertex presentation. Women with high-risk pregnancies, psychiatric illness, or contraindications to massage were excluded. A total of 180 participants were enrolled using convenience sampling and randomly allocated equally into experimental (n=90) and control (n=90) groups.

## Intervention protocol:

The experimental group received effleurage back massage consisting of gentle, circular stroking movements applied to the lumbar area by a trained nurse during uterine contractions. The massage sessions lasted 5 to 8 minutes and were repeated at regular intervals throughout the active labor phase until full cervical dilation. The control group received routine care without any massage intervention.

## Data collection tools:

Data were collected using a structured interview schedule for socio-demographic and obstetric details. Pain intensity was measured before and after the intervention using the Visual Analogue Scale (VAS), a validated 10-point numerical scale. The reliability of the VAS was confirmed by a pilot study, showing a Cronbach's alpha of 0.824.

## Ethical considerations:

The study received approval from the institutional ethics committee. Informed written consent was obtained from all participants. Participant confidentiality was maintained and the right to withdraw from the study at any stage was ensured.

## Data analysis:

Data were analyzed using SPSS software version 25. Descriptive statistics summarized baseline characteristics and pain scores. Paired t-tests assessed within-group differences in pain scores before and after intervention, while unpaired t-tests compared differences between groups. Chi-square tests examined associations between demographic variables and pain outcomes. Statistical significance was set at p < 0.05.

## Results:

Table 1 (see PDF) illustrates the demographic characteristics of the 180 participants, revealing that the majority were aged 31-45 years (66.7%), married (67.5%) and predominantly illiterate (49.2%), with none having education beyond secondary school. Most participants reported a monthly income between ₹10,000-15,000. Table 2 (see PDF) summarizes the comparison of pre- and post-test pain scores across experimental and control groups. A significant reduction was observed in the experimental group's post-test scores, indicating the effectiveness of the intervention. Complementing these findings, Figure 1 (see PDF) presents the mean pain scores pre- and post-test by group. The experimental group showed a notable decrease from 6.58 to 5.55, while the control group's scores slightly increased from 7.34 to 7.44, further reinforcing the positive impact of effleurage massage in reducing labor pain.

## Discussion:

This study provides robust evidence that effleurage massage significantly reduces labor pain among primigravida mothers compared to routine care. The reduction in mean pain scores and the marked shift from severe to moderate/mild pain categories highlight the intervention's effectiveness. Consistent results have been documented by Nori *et al.* (2023), who found non-pharmacological methods-particularly massage-have proven effective and safe for labor pain management [9]. Similarly, Cabral *et al.* (2023) conducted a systematic review and reported that labor massage led to significantly lower pain scores and reduced need for analgesia [10]. Ahmed *et al.* (2021) demonstrated that effleurage massage produces notable reductions in both pain intensity and labor duration among primiparous women [11]. Supporting these results, Pawale *et al.* (2020) in India reported that back massage led to a significant drop in pain scores versus controls [12]. Meta-analytic reviews are in strong agreement. Ranjbaran *et al.* (2017) systematically found that massage-including effleurage-consistently relieve labor pain in first-time mothers [7]. Chang *et al.* (2022) further showed that non-pharmacological coping methods particularly massage, rank among the most effective strategies globally [13]. Pawale and Salunkhe (2021) conducted a quasi-experimental study among 40 primipara mothers and found that those who received back massage during the first stage of labor experienced significantly lower post-test pain scores compared to the control group receiving routine care (P < 0.0001), thereby supporting the analgesic effect of tactile stimulation during contractions [12]. Similarly, a systematic review by Choudhary *et al.* (2021) synthesizing evidence from 10 studies concluded that back massage is an effective, non-pharmacological technique for reducing labor pain, especially in settings where pharmacologic interventions may be limited or culturally resisted [14]. Similarly, a study in Indonesia demonstrated that effleurage reduced severe pain from 70% to 0%, replacing it with mild and moderate pain levels post-intervention with the birthing experience [15]. Furthermore, effleurage was found to alleviate psychological discomforts such as anxiety and sleep disturbance during pregnancy, contributing to overall maternal well-being [16]. A comparative study by Pujiastutik *et al.* (2021) found that although both effleurage and endorphin massages effectively reduced latent-phase labor pain, endorphin massage had a slightly higher pain-reducing effect; nonetheless, effleurage still provided significant relief and was easier to implement in routine care [17]. Furthermore, Bingan (2019) reported a significant reduction in pain levels among latent-phase laboring mothers following effleurage massage in a Palangka Raya health center, reinforcing its effectiveness even in lower-resource community settings [18]. Finally, a 2024 study by Bunga *et al.* showed that a combined approach using warm compresses and effleurage massage significantly reduced pain intensity in mothers during the first active labor phase, highlighting the potential for customizable, non-pharmacological care protocols in maternity wards [19]. These studies align closely with our own results, underscoring effleurage massage's broad applicability, safety and relevance across different maternal care contexts.

Yosepha *et al.* (2019) found that effleurage massage amongst primigravida mothers led to a significant decrease in pain during first-stage labor [20]. Santiasari *et al.* (2018) corroborated these findings, demonstrating better pain outcomes with both effleurage and counter-pressure massage [21]. In Egypt, Abd-Ella (2021) found that effleurage massage led to significant decreases in pain scores on the Visual Analogue Scale [15]. (Retni *et al.* (2023) demonstrated similar benefits in latent-phase labor pain management [22]. Barut *et al.* (2024) found that research interest in effleurage is rising globally and trials consistently show its benefit for labor pain control [23]. Complementing these data, a network meta-analysis in BMC Women's Health (2024) concluded that massage therapy not only reduces pain but improves comfort and satisfaction, supporting routine obstetric use [24]. The strength of this study lies in careful participant matching and detailed outcome measurement, providing strong evidence-consistent with international literature-that effleurage massage is safe, effective and well-suited for integration into routine intrapartum care.
